# The Preferred Route for the Diagnosis and Management of Thyroid Carcinoma Among the General Population in Saudi Arabia

**DOI:** 10.7759/cureus.35043

**Published:** 2023-02-16

**Authors:** Sarah S Aldharman, Danah M Albalawi, Ghadeer Daghistani, Meshari S Almutairi, Sarah A Alharbi, Nahlah f Alreshidi

**Affiliations:** 1 College of Medicine, King Saud Bin Abdulaziz University for Health Sciences, Riyadh, SAU; 2 College of Medicine, University of Tabuk, Tabuk, SAU; 3 College of Medicine, King Abdulaziz University, Jeddah, SAU; 4 College of Medicine, Prince Sattam Bin Abdulaziz University, Alkharj, SAU; 5 College of Medicine, Princess Nourah Bint Abdulrahman University, Riyadh, SAU; 6 Internal Medicine, Hail University, Hail, SAU

**Keywords:** saudi arabia, general population, thyroid carcinoma, management, diagnosis

## Abstract

Background: Papillary thyroid carcinoma (PTC) is the most common type of thyroid cancer. Papillary thyroid microcarcinoma (PTMC) is a specific subgroup of PTC. Given their small size, PTMCs are often asymptomatic and behave benignly. This puts physicians in a challenging situation about how to prevent overdiagnosis and overtreatment of PTMC. This study aimed to assess the preferences regarding the route of PTMC diagnosis and treatment among Saudi Arabia's general population.

Methods: This is a cross-sectional questionnaire-based-study conducted among the general population in Saudi Arabia. The target subjects were the general population of Saudi Arabia both genders and different age groups from various regions of Saudi Arabia (Western, Central, Eastern, Southern, and North). Participants who did not complete the questionnaire or did not agree to participate were excluded. A self-administered questionnaire was distributed on different social media platforms to collect data from different regions. Data analysis was conducted by using Statistical Package for the Social Sciences (SPSS) 24.0 version (IBM Inc., Chicago, USA) statistical software. A Chi-square test was used to compare categorical variables.

Results: A total of 1,428 participants were included. The majority of them were females (64.4%), and most of them were aged between 19 and 25 years. Moreover, we found that 4.8% of the study population had a medical history of thyroid cancer. Our results revealed that more than half of respondents (54.6%) would select surgical operation immediately if they have a thyroid nodule less than 1 cm in maximal diameter, which turns out to be a PTC. The vast majority of participants (90.1%) would prefer to do cytologic confirmation immediately if they have a thyroid nodule less than 1 cm in maximal diameter, which has suspicious characteristics of PTC in neck ultrasound examination. Regarding PTMC operation, 59.8% of responders were more concerned about complications than recurrences. We found that neither age nor gender significantly affects decision-making for management or operative extent for PTC. There was a significant difference between age and decision-making for the diagnosis of suspicious thyroid nodules (p value = 0.041).

Conclusion: Our results concluded that most of the participants preferred to select immediate surgery and cytologic confirmation regarding the management and diagnosis of PTC. More research is advised. The need to inform patients about their disease state and treatment options should be highlighted more.

## Introduction

Over the past few decades, there has been an upsurge in thyroid cancer cases worldwide. In the United States, the incidence of thyroid cancer over the last three decades has tripled, rising from 4.9 to 14.7 per 100,000 individuals in 2011 [1]. The most common type of thyroid cancer is papillary thyroid carcinoma (PTC) which carries an outstanding prognosis with a 97% 10-year overall survival rate when treated [2].

Papillary thyroid microcarcinoma (PTMC) is a specific subgroup of PTC with less than 1 cm diameter. Given their small size, PTMCs are often asymptomatic. Therefore, most PTMCs are discovered by chance in patients either by pathologic examination of thyroid glands removed for benign diseases or by imaging modalities such as neck MRI or thyroid ultrasound. The incidence of PTMC has increased during the past 15 years for a variety of reasons including the increased accuracy of the pathologic thyroid examination and the widespread use of fine needle aspiration (FNA) and thyroid ultrasonography [1].

Most PTMCs behave in a benign manner, which presents clinicians with a dilemma over how to avoid overdiagnosis and overtreatment. The American Thyroid Association's (ATA) new guidelines, as of 2015, advise FNA to be used only when nodules are larger than 1 cm in diameter, even in the presence of sonographic patterns that raise concern. Additionally, they endorse the recommendation of active observation for PTMCs as a viable substitute for immediate surgery [3]. The authors think it is crucial to use shared decision-making. Thus, our study aims to assess Saudi Arabian citizens' preferences regarding the timing of PTMC diagnosis and treatment using an online questionnaire.

A study conducted in Switzerland has found that between 1998 and 2012 there has been a significant increase in papillary thyroid and early-stage tumors with a three- to four-fold parallel increase in thyroidectomy, whereas the mortality rate was mildly decreased [4]. These findings indicate that a significant portion of thyroid cancer cases might be overdiagnosed and overtreated [4]. The 2015 American Thyroid Association (ATA) guidelines now recommend that patients with very low-risk tumors are better to be managed with an active surveillance approach as an alternative to immediate surgery [5]. In Japan, more than half of the patients with low-risk papillary thyroid cancer are on active surveillance [6]. In addition, a study conducted in Canada about patients' choice of active surveillance or surgery for managing low-risk papillary thyroid cancer showed that most patients with low-risk papillary thyroid cancer chose active surveillance over immediate surgery [7]. Patients’ choice was influenced by their concerns about either the progression of papillary thyroid cancer or being on thyroid hormone replacement [7]. However, patients who were not offered the option of active surveillance had undergone surgery immediately as it was the only option that the physicians discussed with them [8]. Hence, in order for the patient to fully understand their diagnosis and management options including active surveillance, shared decision-making about the treatment options is important. Moreover, understanding populations’ perceptions of the diagnosis and management of papillary thyroid microcarcinoma may facilitate the distribution of information to these patients. One study from Korea has analyzed the general population’s preferred timing for the diagnosis and management of thyroid carcinoma [9]. The result showed that most of the participants want an immediate diagnosis of the suspicious thyroid nodules regardless of the size, but participants in their thirties prefer delayed diagnosis for unspecified reasons [9]. Regarding the treatment of PTMC, half of the participants want to have immediate surgery following the diagnosis, while the remaining participants prefer active surveillance. Younger participants (those under 40) tend to choose surgery more than older ones [9]. To date, there is little known about how the general population in Saudi Arabia perceives PTMC and when they think is the optimal timing for the diagnosis and management. Therefore, this study aimed to assess the preferred route for the diagnosis and treatment of PTMC in the general population in Saudi Arabia.

## Materials and methods

This is a cross-sectional study conducted in Saudi Arabia between September 2022 and December 2022. The reach population was the general population of Saudi Arabia from various regions of Saudi Arabia. The study was undertaken through a self-administered questionnaire delivered on different online platforms. The questionnaire was designed using Google forms. Then it was distributed on social media platforms. When the participants fill out the questionnaire, the response was saved automatically. All responses were gathered and transported into a Microsoft Excel file for processing information. Data were analyzed employing Statistical Package for the Social Sciences (SPSS) 24.0 version (IBM Inc., Chicago, USA) statistical software. 

Sample size estimation

The representative sample size required is 385 determined by using the Raosoft sample size calculator available online with a margin error determined as 5%, confidence level determined as 95%, and the population of Saudi Arabia determined as 34,000,00. Additional responses were accepted to overcome any missing data or falsely filled forms. A non-probability convenience sampling technique has been utilized.

Inclusion and exclusion criteria

The study's eligibility criteria included the general population of Saudi Arabia -- both genders and different age groups from various regions (Western, Central, Eastern, Southern, and North). Exclusion criteria applied to those who refused to participate or did not complete the questionnaire.

Data collection tool and procedures

We employed a self‐administered questionnaire which was adapted from a prior study on comparable objectives [3].The questionnaire contains two sections. The first section gathers participants' personal information including age, gender, marital status, education level, monthly household income, and past medical history related to thyroid cancer surgery or history of thyroid cancer surgery. The second section contains three questions asking about the preferred route for the diagnosis and management of PTMC. The questionnaire was distributed electronically using Google forms on various social media platforms such as WhatsApp, Twitter, and Telegram. The questionnaire was designed on Google forms by the authors. The questionnaire link was shared on the previous online social media platforms. When the participants open the link and fill out the questionnaire, their responses are automatically saved. Finally, all the responses were transferred from Google forms to Excel sheets using a feature available on Google forms.

An informed consent form was provided to all the subjects before filling out the questionnaire. Confidentiality was maintained by not employing the participant's name or number or any other identifiers. The ethical approval of the study was obtained before initiating the study. The ethical approval was obtained from the research ethics committee (REC) at the University of Hail (Reference No. H-2022-306).

Statistical analysis

After distributing the questionnaire, they were checked for completeness and any missing information. The online data gathering system ensured that all items must be answered, hence ensuring no incomplete questionnaires were submitted. The collected data were first entered into a Microsoft Excel file. Statistical analysis was conducted using SPSS 24.0 version (IBM Inc., Chicago, USA). We used frequencies and percentages to present categorical data and mean ± standard deviation (SD) to express continuous variables. Categorical variables were compared using the Chi-square test. The p-value <0.05 was deemed significant.

## Results

Characteristics of the study participants

In this study, we included a total of 1,428 respondents. Almost two-thirds of them were females (64.4%), and males were (35.6%). Moreover, we found that more than half of the participants were aged between 19 and 25 years old, and only 1.8% of them were classified into the age group above 55 years old. Regarding the marital status of the study population, we reported that the highest proportion of them were single (53.9%), while 43.6% were married. When we assessed the educational level of respondents, we demonstrated that most of them were Bachelor's degree holders (61.8%). With regard to the geographic distribution of participants, our results found that most of them were from the southern region (26.8%), followed by the western (24.3%), central (18.5%), eastern (18.4%), and northern regions (12%). Our finding revealed that 4.8% of the study population had a medical history of thyroid cancer and 11.2% admitted that their relatives/family member had a medical history of thyroid cancer. The characteristics of the study participants are shown in Table 1.

**Table 1:** Characteristics of the study participants (n=1,428).

Variable	Category	Frequency	Percent (%)
Gender	Male	508	35.6
	Female	920	64.4
Age (in years)	< 18	52	3.6
	19-25	773	54.1
	26-35	277	19.4
	36-45	192	13.4
	46-55	109	7.6
	> 55	25	1.8
Marital status	Single	770	53.9
	Married	623	43.6
	Divorced	29	2
	Widowed	6	0.4
Educational level	Public education	474	33.2
	Bachelor's degree	883	61.8
	Postgraduate degree	71	5
Region	Central	264	18.5
	Southern	382	26.8
	Eastern	263	18.4
	Western	347	24.3
	Northern	172	12
Medical history of thyroid cancer	No	1200	84
	Yes, I am	68	4.8
	Yes, relatives/family member	160	11.2

Preferred timing of operation for thyroid carcinoma, cytologic diagnosis for thyroid carcinoma, and management of thyroid carcinoma

Our results showed that more than half of respondents (54.6%) would undergo a surgical operation immediately if they have a thyroid nodule < 1 cm in maximal diameter, as it turns out to be a PTC, whereas 45.4% of them would like to observe and then proceed to operation if significant changes occur. Furthermore, we found that the vast majority of participants would prefer to do cytologic confirmation immediately if they have a thyroid nodule <1 cm in maximal diameter, which has suspicious features of PTC in cervical ultrasound examination (90.1%). In addition, our results revealed that the highest percentage of participants (59.8%) preferred to do a surgical operation with a low risk of complication, tolerating a little increased risk of recurrence rather than a low risk of recurrence, tolerating a little higher chance of complication. Items related to the preferred timing of operation for thyroid carcinoma, cytologic diagnosis for thyroid carcinoma, and management of thyroid carcinoma are shown in Table 2.

**Table 2:** Preferred route of operation for thyroid carcinoma, cytologic diagnosis for thyroid carcinoma, and management of thyroid carcinoma.

Question	N (%)
1. If you have a thyroid nodule less than 1 cm in maximal diameter, which turns out to be a papillary thyroid carcinoma, what would you do?	
Having an operation immediately	780 (54.6%)
Observing and having an operation only if significant changes occur	648 (45.4%)
2. If you have a thyroid nodule less than 1 cm in maximal diameter, which has suspicious characteristics of papillary thyroid carcinoma in neck ultrasound examination, what would you do?	
Having a cytologic confirmation immediately	1287 (90.1%)
Observing and having a cytologic confirmation only if significant changes occur	141 (9.9%)
3. If you decide to have an operation for papillary thyroid carcinoma, which has generally a low risk of recurrence and mortality, which operation would you prefer to have?	
An operation with a low risk of complication, accepting a little increased risk of recurrence	854 (59.8%)
An operation with a low risk of recurrence, accepting a little increased risk of complication	574 (40.2%)

**Figure 1:**
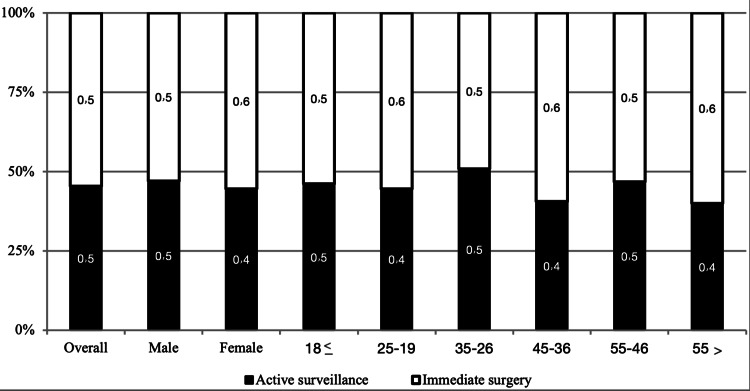
Decision making for management of PTC according to gender and age groups. PTC, papillary thyroid carcinoma

Our results demonstrated that 54.6% of participants decided to do immediate surgery regarding the management of PTC. On the other side 45.4% of preferred the active surveillance. Additionally, our findings showed that neither age nor gender significantly affects decision-making for the management of PTC (p value = 0.346 and 0.327, respectively). Participants above 55 years had a relatively higher tendency to choose immediate surgery than other age groups but without any significant difference. Figure 1 presents decision-making for the management of PTC according to gender and age groups. 

**Figure 2:**
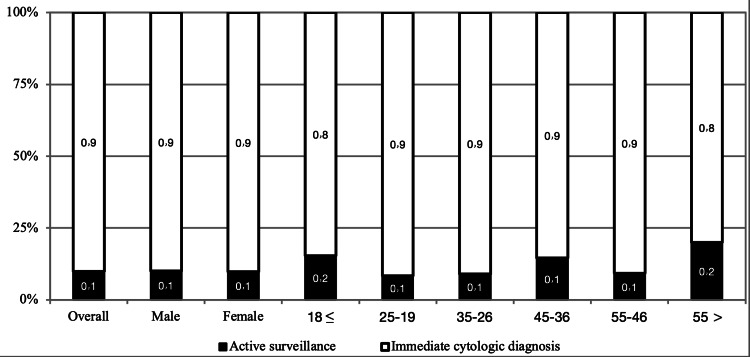
Decision making for a suspicious thyroid nodule according to gender and age groups.

Concerning the diagnosis of a suspicious thyroid nodule, we observed that the majority of respondents tended to do immediate cytologic diagnosis (90.1%). There was a significant difference between age and decision-making for the diagnosis of a suspicious thyroid nodule (p value = 0.041). Respondents above 55 years had a lower tendency for making an immediate cytologic diagnosis than other age groups. Furthermore, we found that age did not significantly affect the diagnosis decision regarding a suspicious thyroid nodule (p value = 0.876). Figure 2 shows decision-making for a suspicious thyroid nodule (S) according to gender and age groups.

**Figure 3:**
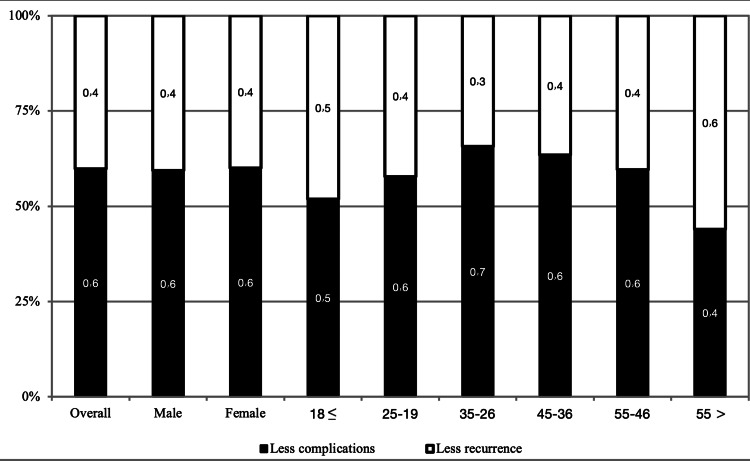
Decision making for the operative extent of PTC according to gender and age groups. PTC, papillary thyroid carcinoma

When we assessed the decision-making for operative extent for PTC, we noted that more than half of the participants would accept less complications of surgery rather than less recurrence of PTC. Gender did not show any significant association with decision-making for operative extent for PTC (p value = 0.839). On the other hand, age showed a relatively higher correlation with decision-making for operative extent for PTC, but without any significant difference (p value = 0.066). Figure 3 shows the decision-making for the operative extent of PTC according to gender and age groups.

## Discussion

In this study, we examined the preferred route for the diagnosis and management of thyroid carcinoma among the general population in Saudi Arabia. Thyroid cancer accounts for more than 90% of all endocrine system malignancies [9]. Significant advancements have been made in the diagnosis and treatment of PTMC. Most researchers concur that PTMCs have a slow disease course in general [10-11]. Saudi Arabia's population census, as well as its economic and cultural structure, have seen significant changes. Thyroid cancer incidence has increased significantly, according to data from the National Cancer Registry [9, 12].

Our study found that more than half of respondents (54.6%) would like to have a thyroid nodule < 1 cm in maximum diameter removed right away. Another survey performed in Korea indicated that 59.5% of respondents wanted it to be removed immediately [3]. Two Japanese studies, however, suggested that active surveillance as the first line of therapy for low-risk PMCs and preclinical low-risk PTMCs can be monitored without rapid surgery and that elderly patients with PTMC may be the best candidates for monitoring [13-14]. Another study in China aimed to examine the management of individuals with suspicious thyroid nodules. It showed that 212 (54.9%) received active surveillance, whereas 174 (45.1%) underwent immediate surgery [15]. Changes to the ATA recommendations for PTC patients have raised the requirement for shared decision-making and personalized treatment programs [16]. Previous research has suggested that non-operative therapy for patients with PTCs less than 2 cm should be utilized with caution. Patients over the age of 45 with PTCs less than 2 cm should have a thyroidectomy [17].

Additionally, our finding reported that the vast majority of participants would prefer to choose cytologic confirmation immediately if they have a thyroid nodule < 1 cm in maximal diameter. This finding was consistent with another study which found that if a suspected thyroid nodule measured less than 1 cm in diameter, 95.7% of respondents wanted a cytological diagnosis [3]. According to an earlier study, 109 patients underwent surgical therapy after being observed for various reasons. The most common reason given was tumor enlargement [18]. To minimize overtreatment of clinically inconsequential PTMCs, new ATA guidelines issued in 2015 propose FNA for nodules more than 1 cm in largest size even with high-suspicion sonographic characteristics [5].

Moreover, our results revealed that the highest percentage of participants (59.8%) selected a surgical operation with a low risk of complication, tolerating a little increased chance of recurrence. Another study found that (53.0%) of participants were more concerned about recurrences than complications. In this study, participants over the age of 55 had a little higher proclivity to select immediate surgery than other age groups, although the difference was not statistically significant. This was contradicted with another Korean study, which found that respondents under the age of 40 are more likely than others to request emergency surgery: 66.7% vs. 32.7% (p = 0.05) [3]. 

Due to the inherent constraints of an online questionnaire survey, our study had some limitations. Young people predominated in responses since only those who have access to the internet could do so. However, we distributed our online questionnaire through different social media platforms to reach the maximum number of people from different regions in Saudi Arabia. Although respondents can obtain the information, they need to make judgments in each case, this information may not be sufficient to fully comprehend the characteristics of PTMCs and does not take into account potential confounding factors that could affect decision-making. Additionally, those with a history of thyroid cancer were included in the study, which may influence the interpretation of the results. Understanding how having thyroid cancer might influence those groups' decision-making can be the focus of a subsequent study with a similar purpose. Our study, the first in the region, examined the relationship between demographic data and the preferred route for thyroid cancer diagnosis and treatment. Understanding these correlations might be useful in the future for focusing educational and awareness campaigns on the appropriate audience. More clinical epidemiological research is recommended to be conducted in Saudi Arabia to establish effective techniques for increasing awareness, screening, early detection, diagnosis, and therapy of papillary thyroid cancer.

## Conclusions

Our findings showed that, when it came to the treatment and diagnosis of PTC, the majority of participants opted to choose urgent surgery and cytologic confirmation. Age and the determination of the diagnosis of a suspicious thyroid nodule varied significantly. Most participants preferred fewer surgical complications to fewer tumor recurrences. Further focused research is recommended.
